# Inhibition of Rho Activity Increases Expression of SaeRS-Dependent Virulence Factor Genes in Staphylococcus aureus, Showing a Link between Transcription Termination, Antibiotic Action, and Virulence

**DOI:** 10.1128/mBio.01332-18

**Published:** 2018-09-18

**Authors:** Anna Nagel, Stephan Michalik, Michel Debarbouille, Tobias Hertlein, Manuela Gesell Salazar, Hermann Rath, Tarek Msadek, Knut Ohlsen, Jan Maarten van Dijl, Uwe Völker, Ulrike Mäder

**Affiliations:** a Interfaculty Institute for Genetics and Functional Genomics, University Medicine Greifswald, Greifswald, Germany; b Biology of Gram-Positive Pathogens, Department of Microbiology, Institut Pasteur and CNRS ERL 3526, Paris, France; c Institute for Molecular Infection Biology, University of Würzburg, Würzburg, Germany; d Department of Medical Microbiology, University Medical Center Groningen, University of Groningen, Groningen, The Netherlands; KUMC

**Keywords:** SaeRS TCS, *Staphylococcus aureus*, antisense transcription, bicyclomycin, proteome, transcription termination

## Abstract

Staphylococcus aureus causes various diseases ranging from skin and soft tissue infections to life-threatening infections. Adaptation to the different host niches is controlled by a complex network of transcriptional regulators. Global profiling of condition-dependent transcription revealed adaptation of S. aureus HG001 at the levels of transcription initiation and termination. In particular, deletion of the gene encoding the Rho transcription termination factor triggered a remarkable overall increase in antisense transcription and gene expression changes attributable to indirect regulatory effects. The goal of the present study was a detailed comparative analysis of S. aureus HG001 and its isogenic *rho* deletion mutant. Proteome analysis revealed significant differences in cellular and extracellular protein profiles, most notably increased amounts of the proteins belonging to the SaeR regulon in the Rho-deficient strain. The SaeRS two-component system acts as a major regulator of virulence gene expression in staphylococci. Higher levels of SaeRS-dependent virulence factors such as adhesins, toxins, and immune evasion proteins in the *rho* mutant resulted in higher virulence in a murine bacteremia model, which was alleviated in a *rho* complemented strain. Inhibition of Rho activity by bicyclomycin, a specific inhibitor of Rho activity, also induced the expression of SaeRS-dependent genes, at both the mRNA and protein levels, to the same extent as observed in the *rho* mutant. Taken together, these findings indicate that activation of the Sae system in the absence of Rho is directly linked to Rho’s transcription termination activity and establish a new link between antibiotic action and virulence gene expression in S. aureus.

## Abstract (precis)

The major human pathogen Staphylococcus aureus is a widespread commensal bacterium but also the most common cause of nosocomial infections. It adapts to the different host niches through a complex gene regulatory network. We show here that the Rho transcription termination factor, which represses pervasive antisense transcription in various bacteria, including S. aureus, plays a role in controlling SaeRS-dependent virulence gene expression. A Rho-deficient strain produces larger amounts of secreted virulence factors *in vitro* and shows increased virulence in mice. We also show that treatment of S. aureus with the antibiotic bicyclomycin, which inhibits Rho activity and is effective against Gram-negative bacteria, induces the same changes in the proteome as observed in the Rho-deficient strain. Our results reveal for the first time a link between transcription termination and virulence regulation in S. aureus, which implies a novel mechanism by which an antibiotic can modulate the expression of virulence factors.

## INTRODUCTION

The Gram-positive bacterium Staphylococcus aureus causes various diseases ranging from skin and soft tissue infections to life-threatening infections such as endocarditis, sepsis, and toxin-mediated diseases (1, 2). It is also a commensal that persistently colonizes the skin and nasal cavity of about 20% of the human population (3, 4). A complex regulatory network mediates the adaptation of S. aureus to the host during colonization and infection. In particular, expression of genes involved in virulence and biofilm formation is tightly controlled by multiple regulatory systems comprising two-component systems, transcription factors, the alternative sigma factor SigB, and small regulatory RNAs (for reviews, see references 5 to 7). In addition, genomic alterations caused by prophage induction contribute to changes in virulence factor production in response to host factors and antibiotics (8–10).

A key regulator of virulence gene expression in staphylococci is the SaeRS two-component system consisting of the membrane-bound SaeS sensor kinase and the cytoplasmic SaeR DNA-binding response regulator (11). The Sae system contains two additional proteins, the SaeP lipoprotein and the SaeQ membrane protein, which modulate its activity by activating the phosphatase activity of SaeS (12). SaeS belongs to the family of so-called “intramembrane-sensing kinases,” which possess an N-terminal domain composed of two transmembrane helices with a short extracytoplasmic linker and lack a specific extracytoplasmic sensory domain (13).

The Sae system responds to multiple stimuli (for a review, see reference 14), including exposure to subinhibitory concentrations of human neutrophil peptides (HNPs), the neutrophil protein calprotectin, or inhibitory concentrations of hydrogen peroxide (15, 16). Induction of the SaeR regulon in a strain producing a constitutively active form of the response regulator WalR suggested that the WalKR two-component system, which is important for cell wall homeostasis, can positively affect the Sae system (17). Although the molecular mechanism is unknown, recent results suggest that this may be an indirect effect through interaction of the SpdC Abi-domain protein with SaeS (18). Studies also reported a cross talk of the Sae system with other global regulators, but to date there is no evidence for any direct regulatory interaction (14). Typically, activation of the Sae system is required for target gene induction, whereas in S. aureus Newman a single amino acid substitution in the transmembrane domain of SaeS leads to strong constitutive kinase activity of SaeS and high constitutive expression of low-affinity target genes (19). Target genes thought to have high-affinity binding sites for phosphorylated SaeR (*hla* and *hlb*) are insensitive to the *saeS* polymorphism, since the basal level of SaeR phosphorylation is sufficient for their transcription (19).

The Sae system controls the expression of approximately 40 genes, most of which encode virulence factors such as adhesins, toxins, and immune evasion proteins (20–27). It particularly activates the production of secreted proteins that are key factors in the pathogenesis of S. aureus infections. Several SaeRS-dependent virulence factors are involved in survival of S. aureus in the bloodstream (by interfering with chemotaxis of immune cells or activation of complement and by lysis of phagocytic cells), dissemination into host tissues, and formation of abscesses (28–30). In addition, the extracellular adherence protein (Eap) and fibronectin-binding proteins (FnbA and FnbB) encoded by SaeR regulon genes are major determinants facilitating invasion of nonprofessional phagocytes such as epithelial and endothelial cells (31).

Gene expression is controlled at multiple levels beyond transcription initiation, one of which is transcription termination, involving two major mechanisms (reviewed in reference 32). In the case of intrinsic termination, which is universal to all bacteria, dissociation of the elongation complex depends directly on the properties of the transcribed RNA sequence. The second mechanism relying on the Rho transcription termination factor coexists with intrinsic termination in the vast majority of bacteria (33). Rho is a homohexameric ring-shaped protein with RNA-dependent ATPase and RNA-DNA helicase activities. Rho loads onto the nascent RNA, translocates 5′ to 3′ by threading the RNA through its central pore until it encounters a paused elongation complex, and then dissociates the RNA polymerase from the DNA and RNA (32). Most of the knowledge on the molecular mechanisms and physiological roles of Rho-dependent termination was gained by studies in Escherichia coli (reviewed in reference 34). Recent work has suggested that the essential role of Rho in E. coli lies in the maintenance of chromosome integrity by controlling potentially deleterious transcription-replication collisions (35). In particular, this involves removal of arrested elongation complexes and prevention of extensive R-loop formation (36–38). Unlike in E. coli and several other Gram-negative bacteria, Rho is dispensable in most Gram-positive bacteria, including Bacillus subtilis and S. aureus (39, 40).

Rho is the target for the antibiotic bicyclomycin (BCM), which specifically binds to Rho and interferes with its movement along the RNA, thereby inhibiting its action (41, 42). It was isolated from Streptomyces sapporonensis in 1972, and its protective effect *in vivo* was shown in experimental mouse infections with clinical E. coli isolates (43). BCM is effective against Gram-negative bacteria, because Rho is essential for their viability (44). BCM was used for treatment of diarrhea in humans in the 1980s (45) and is currently in use in veterinary medicine to treat gastrointestinal tract infections (41). In recent efforts to address the growing problem of antimicrobial resistance by reviving old compounds, its bactericidal potential gained by combination with inhibitors of protein synthesis has been shown in *in vitro* studies (46).

In a large-scale transcriptome study using strand-specific tiling arrays, S. aureus HG001, a derivative of the prototype strain NCTC 8325, was analyzed under multiple experimental conditions covering a broad spectrum of the bacterium’s lifestyles, from optimal *in vitro* growth to interaction with host cells (47). Classification of noncoding RNAs identified in this study revealed a relatively low abundance of antisense RNAs (asRNAs) in the S. aureus wild type, where they overlap only 6% of the coding genes. In line with studies showing that the transcription termination factor Rho plays a major role in suppressing antisense transcription in Gram-negative and Gram-positive bacteria, namely, E. coli (48), B. subtilis (49), and Mycobacterium tuberculosis (50), transcriptome analysis of an S. aureus *rho* deletion mutant revealed a remarkable overall increase in antisense transcription in the absence of Rho (47). The S. aureus *rho* mutant exhibited a massive upregulation of transcript levels, particularly on the antisense strand of protein coding genes. Generation of antisense transcripts resulted from extended transcription beyond mRNA ends and transcription from cryptic low-level promoters. Interestingly, the largest group of antisense transcripts was formed by those detectable only in the *rho* mutant, i.e., initiating from promoters that are cryptic in Rho-proficient S. aureus. Additional transcriptome changes observed under Rho-deficient conditions resulted from altered expression of coding genes. These changes are most likely indirect regulatory effects of Rho inactivation through altered activities of transcriptional regulators.

Based on these observations, the goal of the present study was a detailed comparative analysis of S. aureus HG001 and its isogenic *rho* deletion mutant as well as of the effects of the antibiotic BCM on staphylococcal gene expression. Proteome analysis revealed significant differences in the abundances of several proteins, most notably increased amounts of SaeRS-dependent virulence factors such as extracellular adherence protein (Eap), chemotaxis inhibitory protein (CHIPS), and leukotoxins (HlgBC and LukGH), under Rho-deficient conditions. The *in vivo* relevance of increased virulence factor production in the *rho* deletion mutant was confirmed in a murine model of bacteremia. Inhibition of Rho activity by BCM induced the expression of SaeRS-dependent virulence factor genes, both on mRNA and on protein levels, to the same extent as that observed in the *rho* deletion mutant.

## RESULTS

### Effects of *rho* deletion on the S. aureus proteome.

In a previous study, the impact of Rho deficiency was investigated by comparatively profiling the transcriptomes of S. aureus HG001 and an isogenic *rho* deletion mutant harvested during exponential growth and 4 h after entry into stationary phase in RPMI and tryptic soy broth (TSB) medium (47). This analysis revealed a strong overall increase in antisense transcription in the absence of Rho as well as altered expression levels of many genes, the latter likely due to indirect regulatory effects. In order to explore the differences in the cellular and extracellular proteomes of S. aureus HG001 and the Δ*rho* mutant ST1258 under the same conditions as in the transcriptome study, cells and supernatants were collected from exponential- and stationary-phase cultures in RPMI and TSB medium. In both media, growth of the *rho* mutant was not significantly different from that of the parental strain (see Fig. S1 in the supplemental material). Mass spectrometry analysis resulted in the quantification of 1,347 proteins in the cellular proteome and 999 proteins in the exoproteome. Of these, 76 (cellular proteome) and 167 (exoproteome) proteins showed significantly different abundance (absolute fold change of ≥2) between HG001 and the *rho* mutant under at least one of the four conditions (Table S1).

10.1128/mBio.01332-18.1 FIG S1 Growth of S. aureus HG001 and the Δ*rho* mutant ST1258 in RPMI and TSB medium. Red arrows indicate sampling time points. Download FIG S1, PDF file, 0.2 MB. Copyright © 2018 Nagel et al. 2018 Nagel et al. This is an open-access article distributed under the terms of the Creative Commons Attribution 4.0 International license.

10.1128/mBio.01332-18.3 TABLE S1 Comparative proteome analysis of S. aureus HG001 and the Δ*rho* mutant ST1258 in RPMI and TSB medium. Download Table S1, XLSX file, 0.1 MB. Copyright © 2018 Nagel et al. 2018 Nagel et al. This is an open-access article distributed under the terms of the Creative Commons Attribution 4.0 International license.

Most of the differentially abundant cellular proteins (68/76) displayed increased levels in the mutant. About one-fourth (18/68) of these proteins showed significant differences in levels in both media in at least one of the two growth phases. For seven of these proteins, differential levels were common to all four conditions, among them several virulence factors, namely, coagulase (Coa), the extracellular matrix protein-binding protein (Emp), the LukGH leukocidin F-component (LukH), and the second binding protein of immunoglobulin (Sbi).

In the exoproteome of the *rho* mutant, 131 proteins were present in significantly larger amounts than in the parental strain under at least one condition (Table S1). The vast majority of these proteins were specific for either RPMI (74 proteins) or TSB medium (37 proteins). During growth of S. aureus in RPMI medium, a particularly pronounced impact of Rho deficiency was observed for staphylococcal superantigen-like proteins, 12 of which (Ssl1 to Ssl9, Ssl12, Ssl13, and Ssl14) were at least 3-fold more abundant in the supernatant of the mutant, all except Ssl8 and Ssl9 in both growth phases. The remaining 20 of 131 proteins exhibited significantly larger amounts in the *rho* mutant during growth in both TSB and RPMI medium. The largest overlap (14 proteins) between TSB and RPMI was found in the exoproteome samples generated from stationary-phase cultures, including four proteins (Coa, Sbi, and two proteins of unknown function) with higher levels under all four conditions. One protein of unknown function, SAOUHSC_00622, was present in strongly increased amounts in the *rho* mutant in both proteomic fractions. Strongly decreased levels under all four growth conditions were observed for the serine-aspartate repeat protein SdrD, a cell wall-anchored adhesin, which was the most affected protein during growth in RPMI medium.

**FIG 1:**
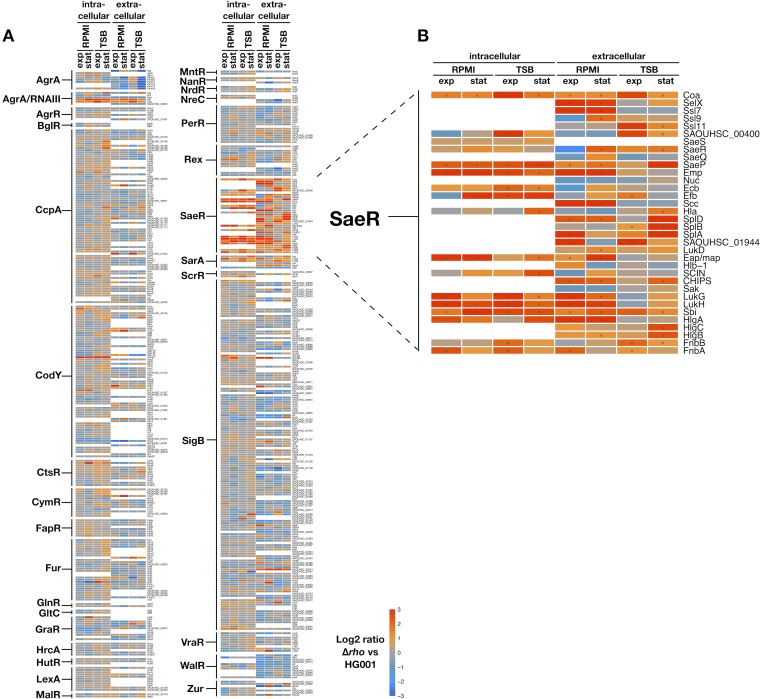
Heat map of protein abundance differences between the Δ*rho* mutant and the parental strain HG001. By mass spectrometry-based proteome analysis, 1,347 proteins were quantified from S. aureus cells and 999 proteins were quantified from the supernatant of HG001 and the Δ*rho* mutant grown in RPMI and TSB medium. (A) Five hundred forty-one of these proteins could be assigned to 30 different transcription factor regulons when including regulons with at least two quantified proteins. Log_2_ ratios (*rho* mutant versus HG001) of mean protein intensities from three independent experiments are displayed. Significantly changed proteins are marked with a cross. Regulons were extracted from RegPrecise (51) and *Aureo*Wiki (52). (B) SaeR-dependent proteins exhibited significantly higher levels in the *rho* mutant.

Changes in gene expression levels in the *rho* mutant can result from (i) transcription downstream of a Rho-dependent termination site (at transcript ends or associated with transcripts not detectable in the wild type) extending into a coding region, (ii) downregulation of sense expression by antisense transcription on the opposite strand, and (iii) indirect regulatory effects resulting from the impact of Rho deficiency on the level and/or activity of transcriptional regulators (47). In order to examine the latter group, we systematically analyzed the extent to which proteome changes in the *rho* mutant can be linked to known regulators (Fig. 1A). Proteins monitored in our analysis substantially covered transcription factor regulons that were extracted from RegPrecise (51) and *Aureo*Wiki (52). However, there was in general no overrepresentation of differentially abundant proteins. The striking exception to this pattern was the group of proteins belonging to the SaeR regulon (Fig. 1B), which were present in significantly larger amounts in the *rho* mutant. In the cellular proteome, 14 SaeRS-dependent proteins exhibited increased amounts under at least one of the four conditions and 27 were more abundant in the exoproteome. Apart from these, proteome changes in the *rho* mutant could not be assigned to distinct regulatory circuits or stress signatures.

### Impact of Rho deficiency on expression of SaeRS-dependent virulence factor genes.

In the comparative proteome analysis of S. aureus HG001 and the Δ*rho* mutant ST1258, 34 of the 39 proteins encoded by known SaeR-regulated genes (Table 1) could be identified. Half of these (17/34) were found exclusively in the exoproteome samples, and one, SaeS, was found in the cellular proteome. The remaining 16 proteins were shared by both proteomic fractions, among them cell surface-associated (FnbA, FnbB, and Sbi) and secreted (Coa, Eap, Ecb, Efb, Emp, Hla, HlgA, LukG, LukH, and SCIN) proteins. The presence of the latter group in the cellular fraction is assumed to be largely due to the retention of secreted proteins at the bacterial cell surface (53).

**TABLE 1:** The SaeR regulon

Locus	Gene name	Protein name	Description	Reference
SAOUHSC_00182	SAOUHSC_00182	SAOUHSC_00182	Unknown function	RegPrecise (51)
SAOUHSC_00192	*coa*	Coa	Coagulase	RegPrecise (51)
SAOUHSC_00354	*selX*	SelX	Staphylococcal enterotoxin-like toxin X	RegPrecise (51)
SAOUHSC_00392	*ssl7*	Ssl7	Staphylococcal superantigen-like protein 7	RegPrecise (51)
SAOUHSC_00394	*ssl9*	Ssl9	Staphylococcal superantigen-like protein 9	RegPrecise (51)
SAOUHSC_00399	*ssl11*	Ssl11	Staphylococcal superantigen-like protein 11	RegPrecise (51)
SAOUHSC_00400	SAOUHSC_00400	SAOUHSC_00400	Unknown function	RegPrecise (51)
SAOUHSC_00402	*lpl3*	Lpl3	Lipoprotein-like protein	RegPrecise (51)
SAOUHSC_00714	*saeS*	SaeS	Sensor histidine kinase	RegPrecise (51)
SAOUHSC_00715	*saeR*	SaeR	Response regulator	RegPrecise (51)
SAOUHSC_00716	*saeQ*	SaeQ	Transmembrane protein SaeQ	RegPrecise (51)
SAOUHSC_00717	*saeP*	SaeP	Lipoprotein SaeP	RegPrecise (51)
SAOUHSC_00816	*emp*	Emp	Extracellular matrix protein-binding protein	21
SAOUHSC_00818	*nuc*	Nuc	Nuclease	24
SAOUHSC_01110	*ecb*	Ecb	Fibrinogen-binding protein-like protein	RegPrecise (51)
SAOUHSC_01114	*efb*	Efb	Extracellular fibrinogen-binding protein	RegPrecise (51)
SAOUHSC_01115	*scc*	Scc	Fibrinogen-binding protein precursor-like protein	RegPrecise (51)
SAOUHSC_01121	*hla*	Hla	Alpha-hemolysin	RegPrecise (51)
SAOUHSC_01938	*splD*	SplD	Serine protease SplD	RegPrecise (51)
SAOUHSC_01939	*splC*	SplC	Serine protease SplC	RegPrecise (51)
SAOUHSC_01941	*splB*	SplB	Serine protease SplB	RegPrecise (51)
SAOUHSC_01942	*splA*	SplA	Serine protease SplA	RegPrecise (51)
SAOUHSC_01944	SAOUHSC_01944	SAOUHSC_01944	Unknown function	RegPrecise (51)
SAOUHSC_01954	*lukD*	LukD	Leukocidin subunit LukD	RegPrecise (51)
SAOUHSC_01955	*lukE*	LukE	Leukocidin subunit LukE	RegPrecise (51)
SAOUHSC_02160	*map2*	Map2	Truncated Map protein	RegPrecise (51)
SAOUHSC_02161	*eap*/*map*	Eap/Map	Extracellular adherence protein	RegPrecise (51)
SAOUHSC_02163	*hlb-1*	Hlb-1	Beta-hemolysin (truncated)	23
SAOUHSC_02167	*scn*	SCIN	Staphylococcal complement inhibitor	RegPrecise (51)
SAOUHSC_02169	*chp*	CHIPS	Chemotaxis inhibitory protein	26
SAOUHSC_02171	*sak*	Sak	Staphylokinase	RegPrecise (51)
SAOUHSC_02241	*lukG*	LukG	Leukocidin subunit LukG	RegPrecise (51)
SAOUHSC_02243	*lukH*	LukH	Leukocidin subunit LukH	RegPrecise (51)
SAOUHSC_02706	*sbi*	Sbi	Second immunoglobulin-binding protein	RegPrecise (51)
SAOUHSC_02708	*hlgA*	HlgA	Gamma-hemolysin subunit HlgA	25
SAOUHSC_02709	*hlgC*	HlgC	Gamma-hemolysin subunit HlgC	RegPrecise (51)
SAOUHSC_02710	*hlgB*	HlgB	Gamma-hemolysin subunit HlgB	RegPrecise (51)
SAOUHSC_02802	*fnbB*	FnbB	Fibronectin-binding protein B	27
SAOUHSC_02803	*fnbA*	FnbA	Fibronectin-binding protein A	27

Altogether, 29 out of 34 SaeRS-dependent proteins exhibited significantly larger amounts in the *rho* mutant under at least one of the four conditions (Fig. 1B and Table S1). Of the remaining five proteins, four exhibited slightly (>1.5-fold) higher protein levels in the mutant and one protein (Nuc) remained unchanged under the conditions of this study, altogether indicating an activation of the Sae system in S. aureus cells lacking Rho activity. A particularly strong increase (>20-fold) was detected for Emp, Efb, LukG, and Sbi in the cellular proteome and for Emp, Scc, SplB, and SAOUHSC_01944 in the exoproteome.

The regulatory components of the Sae system are encoded by the autoregulated *saePQRS* operon (16, 54). Whereas the membrane-localized SaeS sensor kinase and the SaeQ membrane protein were exclusively identified in the cellular or exoproteome samples, respectively, the SaeR response regulator and the SaeP lipoprotein were present in both fractions. The levels of all four proteins were increased in the absence of Rho (Fig. 1), albeit below the 2-fold cutoff in the case of SaeS.

These increased levels of SaeRS-dependent proteins agreed well with the changes exerted by Rho deficiency on the transcriptional level (47), where most of the SaeR regulon members were among the genes that showed significantly higher expression levels in the Δ*rho* mutant. At a stringent threshold (fold change of >2), 30 of the 39 SaeR target genes were found upregulated, 11 of them under all four conditions (exponential and stationary growth phases in the two different media).

### Impact of higher levels of SaeRS-dependent virulence factors in a murine infection model.

**FIG 2:**
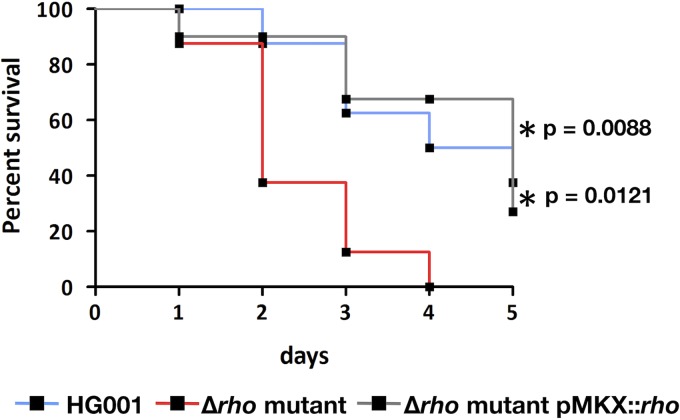
Survival analysis of mice infected with the parental strain HG001, a Δ*rho* mutant, or the *rho* complemented strains. Six- to 8-week-old female mice were infected intravenously with 8 × 10^7^ CFU of HG001 (*n* = 8), the Δ*rho* mutant (*n* = 8), and the complemented strain (*n* = 8). Body weight loss and disease activity index were monitored until day 5 postinfection. Mice with a weight loss of more than 20% of the starting body weight were sacrificed and recorded as dead. The survival rate was significantly different between the parental strain and the Δ*rho* mutant (*P* = 0.0121) and between the Δ*rho* mutant and the complemented strain (*P* = 0.0088). Differences were tested for statistical significance by the Gehan-Breslow-Wilcoxon test.

The Sae system is a major virulence regulatory system of S. aureus and was shown to be important for this bacterium’s virulence after bloodstream infection (55). By expressing a constitutively active version of SaeS from S. aureus Newman (19) in S. aureus USA300 LAC, it was shown previously that constitutive high expression of the SaeR regulon increases virulence in a murine model of bacteremia (56). Therefore, we investigated whether a Rho-deficient S. aureus strain producing higher levels of SaeRS-dependent virulence factors would be more virulent under these *in vivo* conditions. Female BALB/c mice were infected with S. aureus HG001, the Δ*rho* mutant ST1258, or a complementing strain in which *rho* is expressed from a plasmid introduced into strain ST1258. A significant decrease in the survival rate was observed when mice were infected with the mutant compared to the parental strain, whereas virulence of the complemented strain was similar to that of the parental strain (Fig. 2). These results demonstrate the pathophysiological relevance of higher levels of major virulence factors caused by inactivation of the transcription termination factor Rho.

### Effects of Rho inhibition by bicyclomycin on antisense transcription in S. aureus.

BCM is an antibiotic that specifically inhibits Rho, and reducing Rho function by BCM significantly elevates antisense transcription in E. coli (48). Most Gram-positive bacteria are resistant to BCM because Rho is usually not essential in these bacteria. Known exceptions are Micrococcus luteus (57) and M. tuberculosis (50, 58). The latter, however, displays resistane to BCM, which can be explained by a low affinity of its Rho protein for this drug (59). With regard to S. aureus, it was shown previously (40) that an E. coli strain in which the *rho* gene is replaced by the S. aureus *rho* gene is still responsive to BCM addition, indicating that S. aureus Rho is inhibited by BCM. Since S. aureus Rho is sensitive to BCM, we sought to assess for the first time the impact of BCM on the transcriptional profile of a Gram-positive pathogen, which also enabled us to analyze whether all effects observed in the *rho* mutant were directly caused by the lack of Rho’s transcription termination activity.

**FIG 3:**
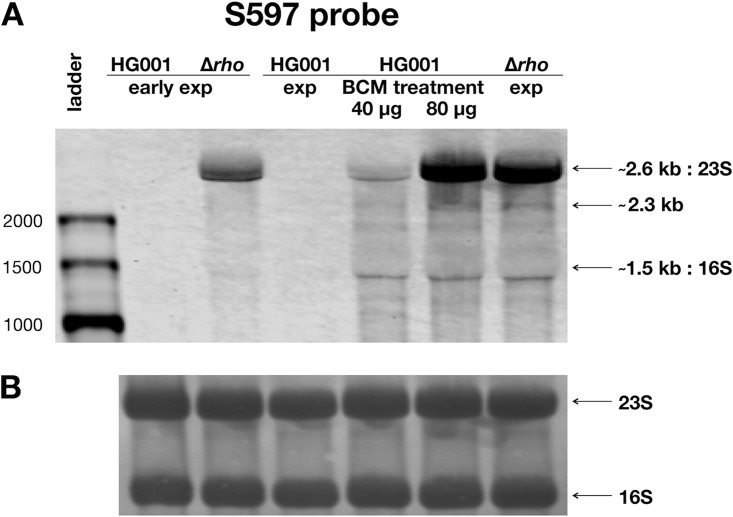
Northern blot analysis of antisense transcription in the absence of active Rho. (A) Transcript levels of asRNA S597 were analyzed by Northern blotting using RNA samples (7 µg per lane) of S. aureus HG001 treated with BCM (40 or 80 µg/ml) and the Δ*rho* mutant. Upon treatment with BCM and in the mutant, a specific band with a size of about 2.3 kb as well as larger transcripts accumulating in the size range of the 23S rRNA were detected. These S597-specific bands were already observed in a previous analysis of Δ*rho* mutant samples (47). Numbers at left are sizes in bases. For the chromosomal location of the S597 gene, see the S. aureus Expression Data Browser at http://genome.jouy.inra.fr/cgi-bin/aeb/viewdetail.py?id=S597_1363050_1363769_1. (B) The methylene blue-stained rRNA bands as control for equal loading of RNA.

We initially compared the effects of *rho* deletion and the impact of BCM on antisense transcription in S. aureus. To this end, HG001 was treated with different concentrations of BCM during exponential growth in RPMI medium. RPMI medium was selected for these experiments because global gene expression profiles of S. aureus cells grown in RPMI medium and human plasma were previously shown to be largely similar (47). BCM was added to the cultures at an optical density at 600 nm (OD_600_) of 0.2, and growth was pursued for 70 min (60). RNA was extracted from HG001 cells harvested at an OD_600_ of 0.2 and grown for 70 min with or without BCM as well as from Δ*rho* mutant (ST1258) cells harvested at an OD_600_ of 0.2 and 70 min later. For Northern blot analysis, asRNAs were selected which exhibited increased levels in the *rho* mutant as revealed by the previous tiling array study (47). Treatment of S. aureus with BCM at a concentration of 20 µg/ml, which reduces Rho function in E. coli without affecting cell growth (57), did not result in increased asRNA levels. However, as exemplified for S597, Northern blot analysis revealed an increase in asRNA levels after treatment with 40 µg/ml of BCM, albeit still significantly lower than the *rho* mutant (Fig. 3). Upon inhibition of Rho activity with 80 µg/ml of BCM, transcript levels in the parental strain reached those observed in the *rho* mutant. Growth of S. aureus was not affected under these conditions.

**FIG 4:**
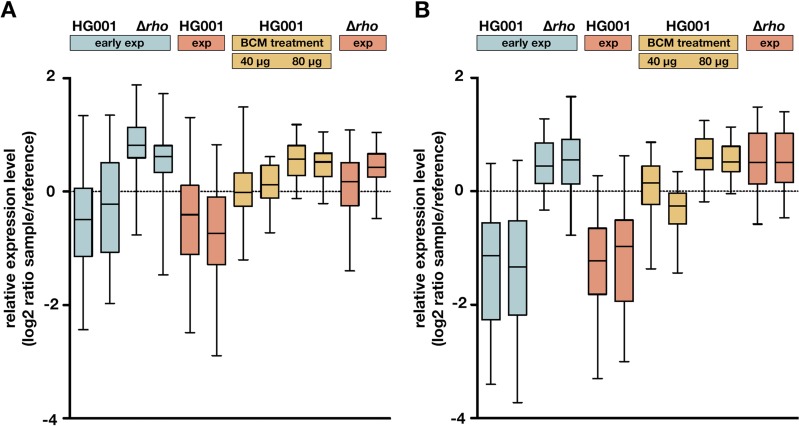
Transcriptome analysis of S. aureus treated with bicyclomycin. Box plots of relative expression levels showing 21 asRNAs of S. aureus identified by Mäder et al. (47) (A) and 39 genes belonging to the SaeR regulon (B). Samples from the parental strain (HG001) and the mutant (*Δrho*) were collected in early exponential growth phase at an OD_600_ of 0.2 (early exp) and 70 min later (exp). At an OD_600_ of 0.2, HG001 cells were treated with 40 or 80 µg/ml BCM, resulting in two additional samples after 70 min (BCM treatment). Expression of antisense RNAs and SaeRS-dependent virulence factor genes increased in the parental strain after treatment with BCM in a dose-dependent manner. Log_2_-transformed relative expression levels are shown for two independent experiments.

To analyze the effects of BCM treatment on antisense transcription at a global level, we compared the transcriptome of the parental strain grown in RPMI medium with or without 40 or 80 µg/ml of BCM to that of the Δ*rho* mutant using Agilent microarrays. Bacteria harvested before BCM treatment at an OD_600_ of 0.2 were included in the analysis. In the previous tiling array study (47), 26 of 145 asRNAs identified in S. aureus HG001 were detected at elevated levels in the *rho* mutant. Of these, 21 asRNAs are represented on the Agilent custom microarray used in this study. The analysis revealed that 14 of these 21 asRNAs exhibited 1.5- to 9-fold-higher levels in the *rho* mutant under the growth condition applied here, which all showed similar or slightly higher levels in the BCM-treated parental strain (80 µg/ml) compared to the *rho* mutant (Fig. 4A and Table S2).

10.1128/mBio.01332-18.4 TABLE S2 Effect of BCM treatment on antisense transcription and the expression of SaeR-regulated genes. Download Table S2, XLSX file, 0.02 MB. Copyright © 2018 Nagel et al. 2018 Nagel et al. This is an open-access article distributed under the terms of the Creative Commons Attribution 4.0 International license.

### Expression of SaeRS-dependent virulence factor genes under conditions of bicyclomycin treatment.

**FIG 5:**
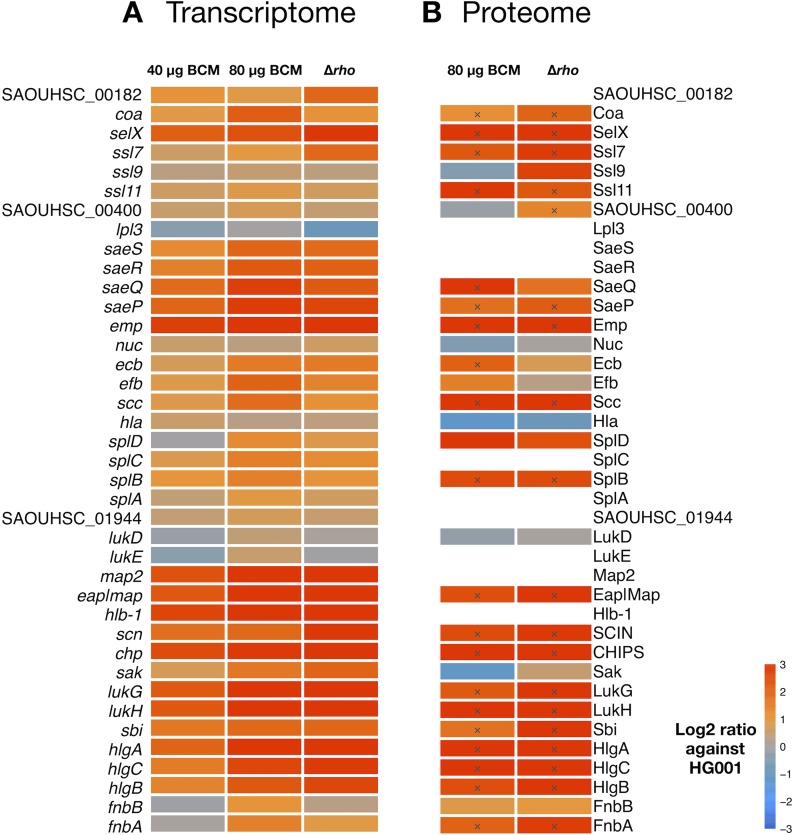
Heat map of bicyclomycin effects on genes and secreted proteins belonging to the SaeR regulon. (A) Expression levels of SaeR-dependent genes were determined by microarray analysis. (B) The corresponding proteins were quantified by mass spectrometry-based analysis after enrichment of proteins from culture supernatants of S. aureus HG001 treated with BCM and the Δ*rho* mutant. HG001 samples were collected in exponential growth phase 70 min after addition of BCM (0, 40, or 80 µg/ml). Untreated mutant samples were collected at the same time point. Log_2_ ratios (BCM-treated HG001 or *rho* mutant versus untreated HG001) of mean expression levels (A) and mean protein intensities (B) from two independent experiments are shown. Significantly changed proteins are marked with a cross.

We then sought to explore if BCM treatment of S. aureus would also result in larger amounts of secreted virulence factors, as would be expected if the effect of Rho on the Sae system is directly linked to its transcription termination activity. Therefore, we analyzed SaeRS-dependent gene expression and the amounts of extracellular proteins after treatment of the parental strain compared to the Δ*rho* mutant. Microarray data revealed higher expression levels of the SaeR regulon genes in the presence of BCM in the parental strain (Fig. 4B and 5A and Table S2), demonstrating activation of the Sae system through inhibition of Rho by this antibiotic. Induction of these genes increased in a dose-dependent manner and reached levels comparable to the *rho* mutant at the highest BCM concentration (80 µg/ml). For individual genes, Rho inhibition by BCM led to even stronger effects than those observed in the mutant (*coa*, *efb*, *fnbA*, *fnbB*, and *scc*), whereas BCM-mediated induction of other genes (*chp*, *emp*, *scn*, *seIX*, *ssl7*, and SAOUHSC_00182) was slightly lower than in the mutant. For example, *chp*, encoding the chemotaxis inhibitory protein CHIPS, was 6- and 8-fold induced by BCM (40 and 80 µg/ml, respectively), but there was a 15-fold expression difference between the Δ*rho* mutant and the untreated parental strain. Samples taken in early exponential phase (OD_600_ of 0.2) served as a control to determine low expression levels of SaeR-regulated genes before BCM treatment (Fig. 4B).

For exoproteome analysis, culture supernatants of S. aureus HG001 grown with or without 80 µg/ml BCM and of the Δ*rho* mutant ST1258 were collected at an OD_600_ of 0.2 before BCM treatment and 70 min later. Proteins from the supernatants were enriched using StrataClean resin beads and analyzed by mass spectrometry. The analysis resulted in the quantification of 776 proteins, of which 41 showed significantly higher abundance in the *rho* mutant than in the parental strain in exponential growth phase (70 min after treatment), whereas only six proteins were present in smaller amounts (Table S3). As depicted in Fig. 5B, higher levels of SaeRS-dependent virulence factors were observed in the BCM-treated HG001 samples than in untreated controls, confirming activation of the Sae system in response to inhibition of Rho. With the exception of SAOUHSC_00400, all proteins present in significantly larger amounts in the *rho* mutant than in the parental strain were induced by BCM treatment (Fig. 5B).

10.1128/mBio.01332-18.5 TABLE S3 Effects of BCM treatment on the extracellular proteome. Download Table S3, XLSX file, 0.03 MB. Copyright © 2018 Nagel et al. 2018 Nagel et al. This is an open-access article distributed under the terms of the Creative Commons Attribution 4.0 International license.

In addition, we compared the effects caused by Rho deficiency and BCM treatment of S. aureus, respectively, by examining all proteins with a significantly and 2-fold-higher abundance in the *rho* mutant than in the parental strain (Table S3). The majority (35/41) of these proteins exhibited increased levels in response to BCM treatment, which were, however, in most cases not as high as in the *rho* mutant. The remaining proteins were not affected by BCM treatment or exhibited slightly reduced levels. The levels of three proteins (SdrD, EsxA, and SAOUHSC_02145) present in significantly smaller amounts in the *rho* mutant were also reduced by BCM treatment of the parental strain. Strikingly, one protein of unknown function (SAOUHSC_00622) showed strongly increased amounts in the *rho* mutant, both in the cellular and in the extracellular fractions, but no effect upon inhibition of Rho function by BCM (Tables S1 and S3), which might point to a specific cellular function of the Rho protein in addition to its transcription termination activity. Proteome analysis of the *rho* complemented strain revealed that ectopic reintroduction of the *rho* gene reverted the levels of this protein to almost those of the parental strain (data not shown), confirming that high SAOUHSC_00622 levels in S. aureus ST1258 were due to Rho deficiency.

## DISCUSSION

The analyses presented here assessed the physiological importance of Rho-dependent transcription termination in S. aureus. The *rho* gene is not essential for viability of S. aureus (40) and can be inactivated without affecting growth under *in vitro* conditions. Nonetheless, a previous study using strand-specific tiling arrays revealed profound changes in the S. aureus transcriptome caused by Rho inactivation, most notably a remarkable overall increase in antisense transcription (47). Transcriptome alterations did not only result from extended transcription beyond mRNA ends or transcription from cryptic low-level promoters but also involved changes in gene expression at the level of transcription initiation, as suggested by the fact that the concerned genes are not preceded by a Rho-dependent termination site. In the present study, we investigated whether these changes can be attributed to the level and/or activity of transcriptional regulators that are influenced by direct effects of Rho deficiency.

In B. subtilis, Rho-controlled transcription was recently shown to affect the activity of two global regulators, Spo0A and SigD, leading to altered expression of genes involved in cell motility, biofilm formation, and sporulation (61). A study on the NusA transcription factor of B. subtilis showed that by controlling read-through transcription at suboptimal intrinsic terminators, NusA also affects global gene expression (62). Depletion of this essential protein caused a substantial increase in antisense transcription and altered the expression of many genes. This included upregulation of the gene encoding the arginine metabolism regulator AhrC by read-through of a NusA-dependent terminator leading to AhrC target gene activation. Another recent study reported a Rho-dependent mechanism in E. coli that is involved in induction of *rpoS*, encoding the general stress response sigma factor (63).

In the present study, the impact of the absence of Rho on the S. aureus proteome was analyzed using cellular and extracellular fractions of S. aureus HG001 and its isogenic Δ*rho* mutant. The analysis showed significant differences in the amounts of several proteins, notably including major virulence factors whose production is controlled by the SaeRS two-component system, a key regulator of virulence gene expression in staphylococci. The SaeR response regulator controls the expression of approximately 40 genes, most of which encode secreted proteins such as adhesins, toxins, and immune evasion proteins (Table 1). In the proteome analysis, 34 of the 39 proteins encoded by known SaeR-regulated genes could be quantified, of which 29 proteins exhibited significantly larger amounts in the *rho* mutant.

The regulatory components of the Sae system are encoded by the *saePQRS* operon (27). Besides the membrane-bound sensor kinase SaeS and the soluble DNA-binding response regulator SaeR, two additional proteins, the lipoprotein SaeP and the membrane protein SaeQ, modulate its activity by activating the phosphatase activity of SaeS (12). A constitutive promoter is responsible for basal levels of SaeS and SaeR, whereas the promoter in front of *saeP* is autoinduced by phosphorylated SaeR (16, 54). The four Sae proteins were found in increased amounts in the proteome of the *rho* mutant, albeit below the 2-fold cutoff in the case of SaeS. However, increased amounts of the regulatory proteins are not assumed to cause an increase in the expression of SaeR target genes (12, 19), but activation at the *saeP* promoter is rather the consequence of higher levels of SaeR phosphorylation in the *rho* mutant. Thus, our data suggest that in S. aureus cells lacking Rho activity, the Sae system is activated, which leads to the observed induction of the SaeR regulon.

The Sae system is known to be induced by different stimuli, in particular host-related signals. Accessory membrane proteins are thought to be involved in sensing external signals, and the N-terminal domain of SaeS, composed of two transmembrane helices with a short extracytoplasmic linker, is responsible for transducing the signals to the kinase domain (13). Since the kinase activity of SaeS appears to respond to the overall conformation of the transmembrane domain, any exogenous or endogenous molecules altering the conformation of this domain can affect phosphorylation of SaeR (64). Accordingly, most of the experimentally investigated intramembrane-sensing histidine kinases are implicated in responding to cell envelope stress (13). For the Sae system, it was previously shown that it is stimulated by constitutive activation of the WalR response regulator involved in cell wall metabolism and autolysis (17). In *S. aur*eus the activity of autolysins is associated with biofilm formation and the release of genomic DNA (65, 66). A recent study showed that the amount of extracellular DNA was decreased in an S. aureus strain lacking both lipase genes (*lip*/*gehA* and *geh*/*gehB*), suggesting that these secreted lipases might affect the integrity of the cytoplasmic membrane (67). Strikingly, in the present study the Geh (or SAL2) protein exhibited higher levels in the *rho* mutant during growth in both media (see Table S1 in the supplemental material). Thus, the signal that triggers activation of the Sae system under conditions of Rho deficiency may be generated by altered lipase activity affecting membrane structure.

The serine-aspartate repeat protein SdrD, a cell wall-anchored adhesin, exhibited strongly decreased levels in the *rho* mutant under all growth conditions analyzed. Strikingly, Kuroda et al. (22) found that *sdrD* and *sdrE* were downregulated upon cefoxitin-mediated activation of the Sae system (see below) in S. aureus N315, presumably in an indirect manner. This strongly suggests that the effect of Rho inactivation on *sdrD* expression observed in our study is linked to the activation of the Sae system and thus reinforces the view that SaeS is activated by deletion of *rho*.

The antibiotic BCM specifically binds to Rho and inhibits its action by preventing ATP turnover and RNA translocation (41, 42). Reducing Rho function by BCM had revealed main physiological roles of Rho in E. coli, including silencing of horizontally acquired genes (68), preventing the formation of DNA double-strand breaks (38), and suppression of pervasive antisense transcription (48). In the Gram-positive bacteria B. subtilis and S. aureus, genome-wide analyses of Rho function were performed using *rho* deletion mutants (47, 49, 61). As mentioned above, these studies demonstrated that a general role of Rho is to suppress or limit bacterial antisense transcription. We now show that inhibition of Rho activity by BCM similarly leads to increased levels of antisense transcripts in Rho-proficient S. aureus cells. More importantly, upon inhibition of Rho activity by BCM, S. aureus produces larger amounts of SaeRS-dependent virulence factors, reaching levels comparable with those observed in the *rho* deletion mutant (Fig. 5). This result indicates that activation of the Sae system under Rho-deficient conditions is directly linked to Rho’s transcription termination activity and establishes a new link between antibiotic action and deleterious virulence gene expression in S. aureus.

Higher levels of SaeRS-dependent virulence factors in the S. aureus mutant lacking Rho activity can be expected to have an impact under *in vivo* conditions. To test this hypothesis, we used a murine bacteremia model and infected mice with S. aureus HG001, the *rho* deletion mutant, and a *rho* complemented strain. Indeed, Rho inactivation significantly increased virulence of S. aureus for mice (Fig. 2). In the present study, we showed that not only inactivation of the *rho* gene but also inhibition of Rho activity by BCM causes increased production of SaeRS-dependent virulence factors, suggesting an impact of the antibiotic BCM on staphylococcal virulence *in vivo*. Efficient inhibition of Rho activity inside the host is beyond question, since it constitutes the basis for BCM treatment of human and animal infections with Gram-negative bacteria (41), in which Rho is essential for viability.

Previous studies have reported effects of antibiotics on the expression of S. aureus virulence factors (reviewed in reference 69). Most *in vitro* data indicate decreased levels of virulence gene expression upon treatment with ribosome-targeted antibiotics (linezolid and clindamycin), which was confirmed by *in vivo* studies (70–74). On the other hand, cell wall-active antibiotics (β-lactams) were shown to increase toxin production (72, 74, 75). Induction of Panton-Valentine leukocidin expression by β-lactam antibiotics interfering with penicillin-binding protein 1 (PBP1) is mediated by the virulence regulators SarA and Rot (76). Inconsistent results were reported with regard to the effects of β-lactam antibiotics on activation of the Sae system. Cefoxitin, a PBP4-selective β-lactam, was shown to activate the Sae system (22), whereas the nonselective oxacillin had no effect on the Sae system (14). Importantly, all these studies investigated the effects of antistaphylococcal agents. In contrast, in the case of BCM an antibiotic effective against Gram-negative bacteria is implicated in the expression of S. aureus virulence factors. This may have clinical implications, not only for patients suffering from mixed infections with different bacterial species but also for patients with a Gram-negative bacterial infection who are carriers of S. aureus.

## MATERIALS AND METHODS

### Bacterial strains and growth conditions.

The following S. aureus strains were used: HG001 (77), the isogenic Δ*rho* mutant ST1258 (47), and the complemented strain ST1258 pMKX::*rho*. Plasmid pMKX is a derivative of the shuttle vector pMK4 (78), allowing inducible gene expression in S. aureus from the xylose-inducible promoter of the Staphylococcus xylosus *xylA* gene, and was constructed in several steps. A 1.7-kb HindIII-BamHI DNA fragment carrying the *S. xylosus xylR* xylose repressor gene and the *xylA* promoter (79) was first purified from plasmid pRB473-XylR and cloned between the corresponding restriction sites of plasmid pUC18 to give plasmid pUCXyl. The insert was sequenced, and plasmid pUCXyl was then used as a matrix to reamplify the 1.7-kb *xylR-pxylA* fragment by PCR to introduce appropriate restriction sites. This fragment was cloned into plasmid pMK4 to give the expression vector pMKX, which was used for gene complementation experiments. Expression of *rho* from the *xylA* promoter was tested in RPMI and brain heart infusion (BHI) media, where it was identical with and without xylose. mRNA levels of strain ST1258 pMKX::*rho* were comparable to expression from the native promoter in the HG001 wild-type strain (see Fig. S2 in the supplemental material).

10.1128/mBio.01332-18.2 FIG S2 Northern blot analysis of *rho* in RNA samples (5 µg per lane) of S. aureus HG001, the Δ*rho* mutant ST1258, and the complemented strain ST1258 pMKX::*rho*. Cells were grown in RPMI and BHI medium with and without 1% xylose until reaching an OD of 0.4. Download FIG S2, PDF file, 0.2 MB. Copyright © 2018 Nagel et al. 2018 Nagel et al. This is an open-access article distributed under the terms of the Creative Commons Attribution 4.0 International license.

Bacteria were cultivated in RPMI (with l-glutamine and without phenol red and HEPES; Gibco Life Technologies, Carlsbad, CA, USA) and tryptic soy broth (TSB; BD, Franklin Lakes, NJ, USA). The cultures were inoculated with an exponentially growing overnight culture to an optical density at 600 nm (OD_600_) of 0.05 and grown with shaking at 37°C. Cells and supernatant were collected in exponential growth phase (TSB, OD_600_ of 0.5; RPMI, OD_600_ of 0.4) and 4 h after entry into stationary phase (Fig. S1).

### Sample preparation for mass spectrometric analysis.

For investigation of cytoplasmic proteins, cells were harvested by centrifugation (5 min, 4°C, 10,000 × *g*), washed once with phosphate-buffered saline (PBS), and frozen in liquid nitrogen. Bacterial cells were resuspended in 25 mM ammonium bicarbonate buffer and disrupted using FastPrep FP120 (5 times for 30 s each, speed 6.5; Thermo Fisher Scientific, Waltham, MA, USA). The culture supernatants were supplemented with bovine serum albumin (BSA) (5 µg/ml), and extracellular proteins were precipitated with trichloroacetic acid (TCA) (80). Protein concentration was determined by using the Bradford assay (Bio-Rad Laboratories, Munich, Germany).

Proteins of supernatants of BCM-treated S. aureus cells were enriched with StrataClean resin beads according to the method of Bonn et al. (80) with minor modifications, namely, using 50 mM Tris buffer (pH 7) instead of TE buffer (50 mM Tris, 10 mM EDTA, pH 7) and determination of peptide concentrations by the Pierce quantitative colorimetric peptide assay (Thermo Fisher Scientific).

Tryptic digest and peptide purification were performed as previously described (81) using a C_18_ ZipTip column (Millipore, Billerica, MA, USA) with a loading capacity of 5 μg. Before subjecting the samples to mass spectrometry, HRM spike-in mix (Biognosys AG, Schlieren, Switzerland) was added.

### Mass spectrometric measurements and data analysis.

Tryptic peptides were separated by reversed-phase liquid chromatography (Dionex Ultimate 3000 system; Thermo Fisher Scientific) coupled online to electrospray ionization mass spectrometry using a Q Exactive mass spectrometer (Thermo Fisher Scientific). The instrumental setup and the data independent acquisition (DIA) method were used according to the method of Michalik et al. (82) with slight modifications (Table S4).

10.1128/mBio.01332-18.6 TABLE S4 Parameters for mass spectrometry and data analysis. Download Table S4, PDF file, 0.4 MB. Copyright © 2018 Nagel et al. 2018 Nagel et al. This is an open-access article distributed under the terms of the Creative Commons Attribution 4.0 International license.

The ion library for DIA data analysis was constructed based on a Comet database search using an S. aureus protein fasta file from *Aureo*Wiki (http://aureowiki.med.uni-greifswald.de/Main_Page) comprising 2,852 protein entries in a target-decoy approach using trypsin/P digest rule with number of tolerable termini (NTT) 2, ±30 ppm (MS1), and 0.01-Da fragment mass tolerance as well as static (sample preparation-dependent carbamidomethylation; 57.021464) and variable (methionine oxidation; 15.9949) modifications. For library construction, previously published (82) and newly generated (Table S5) data sets were acquired on a Q Exactive mass spectrometer (Thermo Fisher Scientific).

10.1128/mBio.01332-18.7 TABLE S5 Additional data set for spectral library preparation. Download Table S5, PDF file, 0.5 MB. Copyright © 2018 Nagel et al. 2018 Nagel et al. This is an open-access article distributed under the terms of the Creative Commons Attribution 4.0 International license.

The results were processed using the Trans-Proteomic Pipeline (TPP; version 4.8.0 philae) (83) including PeptideProphet and iProphet (84, 85) (minimal peptide length, 7 amino acids). A global protein false-discovery rate (FDR) of 1% was set using MAYU (86). The filtered peptide spectrum matches were used to generate the ion library in Spectronaut (v11.0.15038.14.27660 [Asimov]; Biognosys AG, Schlieren, Switzerland) with the following settings: *m/z* mass range of 300 to 2,000, 6 to 10 fragments per peptide, removal of fragments smaller than 3 amino acids, no segmented regression, and a minimum root mean square error of 0.5. The constructed library consists of 2,154 proteins with 38,570 tryptic peptides.

The analysis of the DIA data (82) was carried out using Spectronaut (v11.0.15038.14.27660 [Asimov]; Biognosys AG, Schlieren, Switzerland) with the settings specified in Table S4. The reported tandem mass spectrometry (MS^2^) areas of ions were analyzed in R (version 3.4.3). The ion intensities were median-median normalized and subsequently analyzed protein-wise with at least 2 assays/ions per protein using a Wilcoxon rank sum test against a fold change of 2 against the control sample with multiple test correction using the Benjamini and Hochberg algorithm. Proteins with an adjusted *P* value below 0.05 were assumed to be significantly regulated. All plots were generated in R using the packages listed in Table S4.

### *In vivo* infection experiments. (i) Ethics statement.

All of the animal studies were approved by the local government of Franconia, Germany (approval number 55.2-2532-2-155), and performed in strict accordance with the guidelines for animal care and experimentation of the German Animal Protection Law and directive 2010/63/EU of the European Union (EU).

### (ii) Infection inoculum.

Overnight cultures of *S. aureu*s strains HG001, ST1258, and ST1258 pMKX::*rho* in BHI medium were diluted to a final OD_600_ of 0.05 in 50 ml fresh BHI medium and grown for 3.5 h at 37°C. After centrifugation, cell pellets were resuspended in BHI with 20% glycerol, aliquoted, and stored at −80°C. For the generation of *in vivo* infection inocula, aliquots were thawed and washed twice with PBS. The desired bacterial concentration (8 × 10^7^ CFU/100 µl) was then adjusted based on the respective OD_600_ values. A sample of the infection inoculum was plated on TSB agar plates in order to control the infection dose.

### (iii) Intravenous S. aureus infection model.

Female BALB/c mice (8 per group, 6 weeks old; Janvier Labs, Le Genest-Saint-Isle, France) were intravenously infected with 8 × 10^7^ CFU of bacteria via the tail vein. The mice were housed in individually ventilated cages under normal diet in groups of five throughout the experiment with *ad libitum* access to food and water. During infection, mice were scored twice a day, and the severity of infection (score; see Table S6) was determined accordingly. If a mouse reached the humane endpoint with a score of 20 points, this mouse was sacrificed and removed from the experiment. In this case, the mouse was regarded as dead beginning with this time point in the survival curve (Fig. 2). Significant difference in the survival of the three groups of mice was determined with the Gehan-Breslow-Wilcoxon test (software: GraphPad Prism 5.0).

10.1128/mBio.01332-18.8 TABLE S6 Clinical score determination to assess severity of infection. Download Table S6, PDF file, 0.3 MB. Copyright © 2018 Nagel et al. 2018 Nagel et al. This is an open-access article distributed under the terms of the Creative Commons Attribution 4.0 International license.

### Transcriptome analysis.

RNA was prepared by acid-phenol extraction after mechanical cell disruption as described previously (49). The quality of the RNA was assessed by means of an Agilent 2100 Bioanalyzer (Agilent Technologies, Santa Clara, CA, USA) according to the manufacturer’s instructions.

For Northern blot analysis, 7 µg of total RNA per sample was separated under denaturing conditions in a 1% agarose gel and transferred onto a positively charged nylon membrane (Roche Pharma AG, Grenzach-Wyhlen, Germany) by vacuum blotting. After cross-linking (120 mJ/cm^2^; Stratalinker UV Crosslinker 1800; Stratagene, CA, USA), hybridization was performed as described previously (87). A biotin-labeled RNA probe was generated by *in vitro* synthesis using a T7 RNA polymerase and Bio-16-UTP (Life Technologies, Carlsbad, CA, USA). PCR synthesis of the templates was performed using the following oligonucleotides: TTAATTTATTAATTTATTTC (SA_01432_AS_for) and CTAATACGACTCACTATAGGGAGACGTATCTCTTTGCTCATCGT (SA_01432_AS_T7rev). Detection of the biotin-labeled probe on the membrane with IRDye 800CW streptavidin (Li-Cor Biosciences, Lincoln, NE, USA) was performed with the Odyssey CLx imager according to the instructions of the manufacturer (Li-Cor Biosciences, Lincoln, NE, USA).

For microarray analysis, 5 µg of total RNA (two biological replicates per condition) was subjected to cDNA synthesis. Synthesis and fluorescence labeling of cDNA followed a strand-specific method using the FairPlay III microarray labeling kit (Agilent Technologies, Santa Clara, CA, USA) and actinomycin D (Calbiochem, Merck KGaA, Darmstadt, Germany) (88). The samples were labeled with Cy5, and a reference pool containing equal amounts of RNA from each sample was labeled with Cy3. Seventy-five nanograms of the two cDNAs were hybridized together to the microarray according to Agilent’s hybridization, washing, and scanning protocol (two-color microarray-based gene expression analysis, version 5.5). Data were extracted and processed using Feature Extraction software (version 11.5.1.1). For each gene, the median of the individual probe ratios was calculated. For data analysis, Genedata Analyst software (Genedata AG, Switzerland) was used.

### Data availability.

The microarray data set is available from NCBI's Gene Expression Omnibus (GEO) database (accession number GSE112980). Mass spectrometry data were deposited in the MassIVE database (https://massive.ucsd.edu/ProteoSAFe/static/massive.jsp?redirect=auth) and can be accessed via accession number MSV000082270.
