# Supplementary figures and images for: Inhibition of Rho Activity Increases Expression of SaeRS-Dependent Virulence Factor Genes in Staphylococcus aureus, Showing a Link between Transcription Termination, Antibiotic Action, and Virulence
Source: mBio. 2018 Sep 18;9(5):e01332-18. doi: 10.1128/mBio.01332-18 (PMC6143737; doi:10.1128/mBio.01332-18)

RPMI

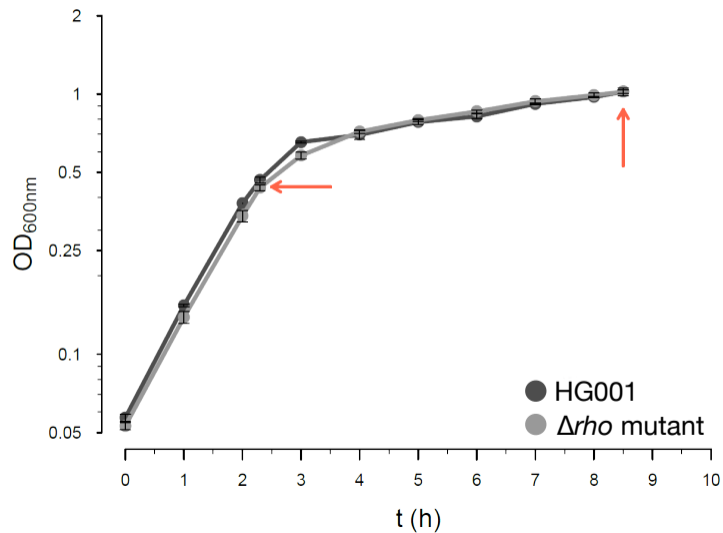

TSB

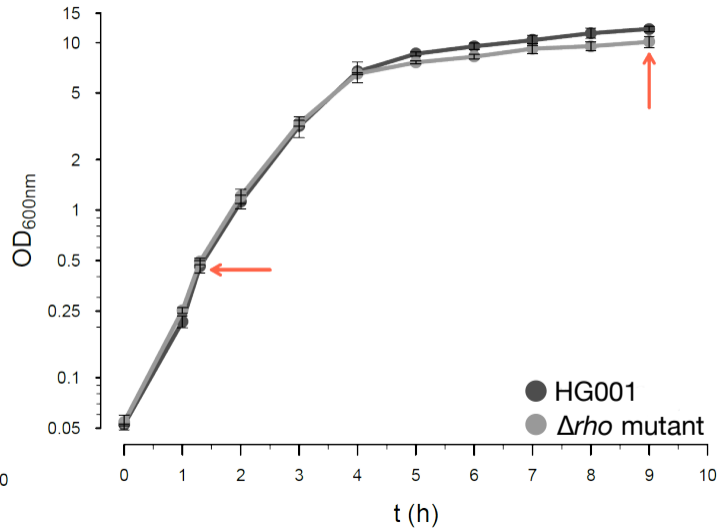

Supplement: FIG S1 [file mbo004184073sf1.pdf]

RPMI

BHI

HG001

 $\Delta rho$  $\Delta rho$   
pMKX::*rho*

HG001

 $\Delta rho$  $\Delta rho$   
pMKX::*rho*

M

-

+

-

+

-

+

-

+

-

+

-

+

1 % Xylose

2000  
1500  
1000  
800  
600  
400 $\rho$ 

23S

16S

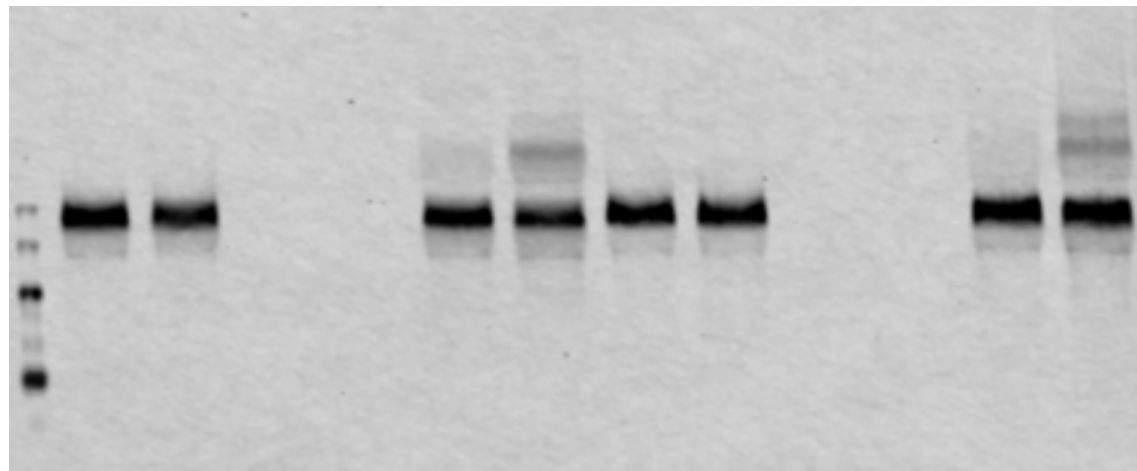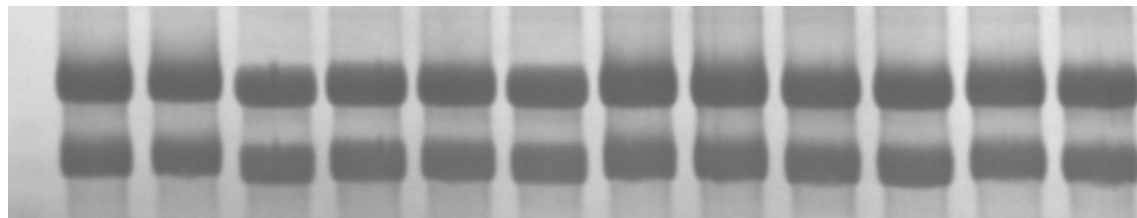

Supplement: FIG S2 [file mbo004184073sf2.pdf]
