# Supplementary material for: Inhibition of Rho Activity Increases Expression of SaeRS-Dependent Virulence Factor Genes in Staphylococcus aureus, Showing a Link between Transcription Termination, Antibiotic Action, and Virulence
Source: mBio. 2018 Sep 18;9(5):e01332-18. doi: 10.1128/mBio.01332-18 (PMC6143737; doi:10.1128/mBio.01332-18)
Supplement: TABLE S4 [file mbo004184073st4.pdf]

Table S4A. Parameter for mass spectrometry analysis.

**Reversed phase liquid chromatography (RPLC)**	
Instrument	Ultimate 3000 RSLC (Thermo Scientific)
Trap column	75 µm inner diameter, packed with 3 µm C18 particles (Acclaim PepMap100, Thermo Fisher Scientific , Waltham, MA, USA)
Analytical column	Accucore 150-C18, (Thermo Fisher Scientific , Waltham, MA, USA)<br>25 cm x 75 µm, 2.6 µm C18 particles, 150 Å pore size
Buffer system	binary buffer system consisting of 0.1% acetic acid, 5% ACN (buffer A) and 100% ACN in 0.1% acetic acid (buffer B)
Flow rate	300 nl/min
Gradient	linear gradient of buffer B from 2% up to 25%
Gradient duration	120 min
Column oven temperature	40 °C
**Mass spectrometry (MS/MS)**	
Instrument	Q Exactive^{TM} mass spectrometer (Thermo Fisher Scientific , Waltham, MA, USA)
Operation mode	data-independent
**Full MS**	
MS scan resolution	35,000
AGC target	5×10^6
Maximum ion injection time for the MS scan	120 ms
Scan range	400 to 1220 m/z
Spectra data type	profile
**dd-MS2**	
Resolution	35,000
MS/MS AGC target	3×10^6
Maximum ion injection time for the MS/MS scans	auto
Spectra data type	profile
Selection for MS/MS	1
Isolation window	Table S4B
Fixed first mass	-
Dissociation mode	higher energy collisional dissociation (HCD)

Table S4B. Isolation windows for data independent acquisition analysis.

Window Number	Range	Window Size
1	400-430	30
2	428-459	31
3	457-483	26
4	481-506	25
5	504-531	27
6	529-554	25
7	552-576	24
8	574-600	26
9	598-624	26
10	622-650	28
11	648-676	28
12	674-704	30
13	702-735	33
14	733-771	38
15	769-810	41
16	808-856	48
17	854-914	60
18	912-1000	88
19	998-1220	222

Table S4C. Spectronaut^{TM} parameters used for analysis.

Parameter Group	Parameter	Setting
Calibration	Calibration Mode	Automatic
	IRT Calibration Strategy	Non-linear iRT calibration
Identification	Pvalue Estimator	Kernel density estimator
	Precurser Qvalue Cutoff	0.001
Workflow	Profiling Strategy	iRT profiling
	Profiling Row Selection	Minimum Qvalue Row Selection
	Profiling Row Selection – Qvalue Threshold	0.001
	Profiling Traget Selection	Profile only non-identfied Precursor
	Profiling Traget Selection – Identification Criterion	Qvalue
	Profiling Target Selection – Threshold	0.0001
	Carry-over exact Peak Boundaries	TRUE
	Unify Peptide Peaks	FALSE
Quantification	Inference Correction	TRUE
	Quantity MS-Level	MS2
	Quantity Type	Area
	Data Filtering	Qvalue percentile
	Percentile	Biological replicate coverage depending (e. g. 3vs3 = 0.5; 3vs3vs3vs3 = 0.125)
	Cross Run Normalization	FALSE
XIC Extraktion	XIC RT Extraction Window	Dynamic
	Correction Factor	1

Table S4D. R packages used for data analysis.

Package	Autor	Title	Version
data.table	Matt Dowle [aut, cre], Arun Srinivasan [aut], Jan Gorecki [ctb], Tom Short [ctb], Steve Lianoglou [ctb], Eduard Antonyan [ctb]	Extension of 'data.frame'	10.10.4-1
dplyr	Hadley Wickham[aut, cre], Romain Francois [aut], Lionel Henry [aut], Kirill Müller [aut], RStudio [cph, fnd]	A Grammar of Data Manipulation	0.7.4
factoextra	Alboukadel Kassambara [aut, cre], Fabian Mundt [aut]	Extract and Visualize the Results of Multivaraite Data Analyses	1.0.5
factoMineR	Francois Husson, Julie Josse, Sebastien Le, RStudio [cph]	Multivariate Exploratory Data Analysis and Data Mining	1.38
ggplot2	Hadley Wickham[aut, cre], Winston Chang [aut], RStudio [cph]	Create Elegant Data Visualisations Using the Grammar of Graphics	2.2.1.9000
ggrepel	Kamil Slowikowski [aut, cre], Jean-Oliver Irisson [ctb], Saulius Lukauskas [ctb], Alicia Schep [ctb], Zhian N Kamvar [ctb], Sean Hughes [ctb], Pierre Gramme [ctb]	Replusive Text and Label Geoms for 'ggplot2'	0.7.0
reshape	Hadley Wickham[aut, cre]	Flexibly Reshape Data	0.8.7
stringr	Hadley Wickham[aut, cre, cph], RStudio [cph]	Simple, Consistent Wrappers for Common String Operations	1.2.0
