# Supplementary material for: Inhibition of Rho Activity Increases Expression of SaeRS-Dependent Virulence Factor Genes in Staphylococcus aureus, Showing a Link between Transcription Termination, Antibiotic Action, and Virulence
Source: mBio. 2018 Sep 18;9(5):e01332-18. doi: 10.1128/mBio.01332-18 (PMC6143737; doi:10.1128/mBio.01332-18)
Supplement: TABLE S5 [file mbo004184073st5.pdf]

Table S5. Additional data set for library preparation.

S. aureus **strain**	**Sample material and cultivation conditions**	**Sampling points**	**Biological replicates**	**Fractionation**	**MS instrument type**	**Number of raw files**
HG001,<br>ST1258,<br>ST1258 pMKX,<br>ST1258 pMKX::*rho*	bacteria from cultivation<br>in RPMI and TSB medium	exponential growth<br><br>stationary growth	3	non-fractionated	Q Exactive™	48
HG001,<br>ST1258	culture supernatants from cultivation<br>in RPMI and TSB medium	exponential growth<br><br>stationary growth	3	non-fractionated	Q Exactive™	24
HG001	bacteria from cultivation in chemically defined medium CDM under different stress conditions<br><br>- heat shock 42 °C and 48 °C<br>- 300 µM 2,2´-bipyridyl<br>- 2 mM diamide,<br>- 2 mM H_2O_2,<br>- 0.12 µM mupirocin,<br>- 10 mM methyl viologen,<br>- 0.3 µg/ml vancomycin	exponential growth as control<br><br>1 h after stress	1	non-fractionated	Q Exactive™	16
HG001	culture supernatants from cultivation<br>in RPMI medium<br><br>samples were filtrated or centrifuged using TCA precipitation compared with StrataClean™ Resin beads	exponential growth	1	non-fractionated	Q Exactive™	10
