# Supplementary material for: Inhibition of Rho Activity Increases Expression of SaeRS-Dependent Virulence Factor Genes in Staphylococcus aureus, Showing a Link between Transcription Termination, Antibiotic Action, and Virulence
Source: mBio. 2018 Sep 18;9(5):e01332-18. doi: 10.1128/mBio.01332-18 (PMC6143737; doi:10.1128/mBio.01332-18)
Supplement: TABLE S6 [file mbo004184073st6.pdf]

Table S6: Clinical score determination to assess severity of infection.

**Observation**	**Score points**
**I Body weight**	
- No change	0
- Loss of body weight in % = score points; e.g. loss of body weight 8% = 8 points	1-20
- Loss of body weight ≥20%	20
**II General conditions**	
**Fur**	
- Shining	0
- Mat	2
- Ruffeled	4
**Eyes**	
- Clear and clean	0
- Unclean and sticky, closed or semi-closed	3
**Posture**	
- Normal	0
- Hunched posture	10
- Massive hunched posture	20
**Clinical complications**	
- Tension, paralysis, tremor	20
- Breath noises	20
- Animal feels cold	20
**III Motility**	
- Spontaneous (normal behaviour, social contacts)	0
- Spontaneous but reduced	1
- Moderately reduced activity	2
- Motility only after stimulation	5
- Isolation, lethargy, coordination disorders	10
- Self-amputation, aggression	20
**IV Respiration**	
- Breathing normal	0
- Breathing slightly changed	1
- Accelerated breathing + 30% (tachypnoea)	10
- Very accelerated breathing +50%	20
**Rating, measures**	**Sum score points**
Severity level 0 = no burden on animals, animals healthy	0-3
Severity level 1 = low burden on animals, low sickness, animals are observed	4-9
Severity level 2 = moderate burden on animals, moderate sickness, animals are carefully observed	10-15
Severity level 3 = moderate to severe burden on animals, moderate to severe sickness, animals are carefully observed, abort of experiment if necessary	16-20
Severity level 4 = severe burden on animals, animals moribund, implementation of humane endpoint, abort of experiment	20 or higher
